# Highly efficient graphene terahertz modulator with tunable electromagnetically induced transparency-like transmission

**DOI:** 10.1038/s41598-023-34020-2

**Published:** 2023-04-24

**Authors:** Myunghwan Kim, Seong-Han Kim, Chul Kang, Soeun Kim, Chul-Sik Kee

**Affiliations:** 1 Division of Applied Photonics System Research, Advanced Photonics Research Institute, Gwangju Institute of Science and Technology, Gwangju, 61005 South Korea; 2 Optical Packaging Research Section, Electronics and Telecommunications Research Institute (ETRI), Gwangju, 61012 South Korea

**Keywords:** Applied physics, Electronics, photonics and device physics, Optical physics

## Abstract

Graphene-based optical modulators have been extensively studied owing to the high mobility and tunable permittivity of graphene. However, weak graphene-light interactions make it difficult to achieve a high modulation depth with low energy consumption. Here, we propose a high-performance graphene-based optical modulator consisting of a photonic crystal structure and a waveguide with graphene that exhibits an electromagnetically-induced-transparency-like (EIT-like) transmission spectrum at terahertz frequency. The high quality-factor guiding mode to generate the EIT-like transmission enhances light-graphene interaction, and the designed modulator achieves a high modulation depth of 98% with a significantly small Fermi level shift of 0.05 eV. The proposed scheme can be utilized in active optical devices that require low power consumption.

## Introduction

The terahertz (THz) spectral range of 0.1–10 THz is an important frequency band owing to its potential applications in various fields, such as security imaging, wireless high-speed communications, and biomedical diagnostics^1–3^. Over the past two decades, THz source generation and detection have achieved substantial progress and revitalized the development of THz technologies^4^. Recently, there has been a rapid development in the THz metamaterial devices^5–8^. However, more research on advanced components in the THz regime is still required. In particular, THz optical modulators, which are key devices for actively controlling THz signals, are crucial in THz communications and imaging^9–12^. Although types of THz optical modulators based on semiconductor materials have been suggested^13,14^, their modulation depths are not sufficient high.

Recently, graphene has garnered significant attention owing to its several exceptional properties, such as high thermal conductivity, remarkable carrier mobility, and wide optical bandwidth^15–17^. In particular, the optical property of graphene can be easily controlled by the gate-voltage^18^. This tunability enables the application of graphene in optical modulators as an active layer. The high carrier mobility on the order of 10^6^ cm/Vs allows fast responses to electromagnetic fields. In addition, cost-effective graphene-based modulators can be realized using the chemical vapor deposition (CVD) method. In recent, a high-quality large-area graphene single layer grown by CVD have been reported in several studies ^19–22^. However, suspended single-layer graphene has a negligible absorption of 2.3% for normal incidence, and the tunable effect is not sufficiently strong for drastic variations in absorption, transmission, or reflection owing to the low light-graphene interaction, which is a significant obstacle in achieving high modulation performance.

To improve light-graphene interaction, graphene plasmons have been introduced^23,24^. In the THz and mid-infrared regions, graphene supports surface plasmons, and the properties of the plasmonic modes can be tuned by adjusting the Fermi level. Several types of optical modulators that utilize graphene plasmons and metamaterials have been reported and they exhibit high modulation depth^25–33^. However, high-quality (high-mobility) graphene is required to induce high quality factor (Q-factor) plasmonic resonance. In addition, high Fermi level graphene doping (> 0.4 eV) is required to achieve high modulation performance, which increases the power consumption. Another approach to increasing the light-graphene interaction is adopting the epsilon-near-zero (ENZ) effect^34,35^. When the permittivity of graphene is approximately zero, the electric field is highly confined within the graphene layer, thereby increasing absorption. However, the ENZ effect in graphene is yet to be experimentally demonstrated and is highly debated^36^. Inserting a graphene layer in a resonator supporting a high Q-factor can enhance the light-graphene interaction^37–40^. For example, optical modulators with the graphene layer placed in photonic crystal (PC) structures or ring resonators that support high Q-factor resonance have been suggested for the optical communication wavelength region. However, studies on optical modulators combining graphene and high Q-factor resonators in the THz frequency band are insufficient.

Here, we numerically demonstrate a low-voltage, high-modulation depth optical modulator by inserting two graphene layers in a structure supporting electromagnetically induced transparency (EIT)-like transmission. EIT is a transmission phenomenon with a very narrow band due to destructive quantum interference^41^. Although realizing EIT is difficult owing to strict experimental conditions, an EIT-like spectral response can be obtained by coupling two resonators^41–44^. We demonstrate that the high Q-factor guiding mode to generate EIT-like transmission enhances the light-graphene interaction, and the transmission can be drastically modified by a negligible Fermi level shift. The proposed modulator achieves a high modulation depth of ~ 98.2% with a Fermi level shift of 0.05 eV. Therefore, we infer that the proposed modulator is highly desirable in many fields, such as in THz imaging and communications. All simulations were conducted using the finite-element-method (COMSOL Multiphysics software).

## Model and principle

**Figure 1:**
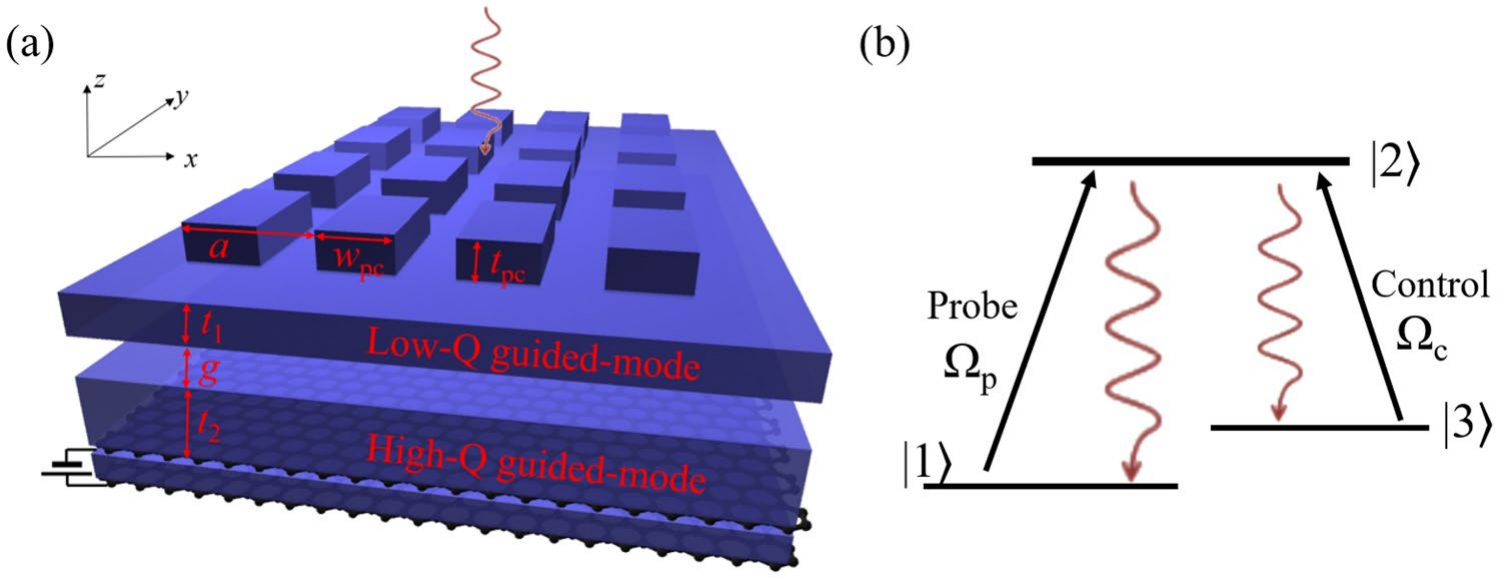
(**a**) Schematic illustration of a graphene-based optical modulator. The modulator comprises a 2D PC and two SiO_2_ waveguides separated by an air gap; in addition, two graphene layers are placed on the bottom waveguide with significantly thin SiO_2_ gap. (**b**) Typical three-level Λ-scheme for EIT.

Figure 1a presents a schematic of the proposed graphene-based optical modulator comprising a two-dimensional (2D) rod-type PC and two waveguides (*W*_top_ and *W*_bot_) with a gap. The PC comprising square-shaped pillars of width *w*_pc_ and height *t*_pc_ is placed on the top waveguide. The refractive indices of SiO_2_ and the background material are assumed to be *n*_SiO2_ = 2 and *n*_b_ = 1, respectively. The refractive index of SiO_2_ varies from 1.953 to 2.108 in the THz region, depending on the deposition method^45,46^. The imaginary part of the refractive index of SiO_2_ is negligible because it is much smaller than that of the refractive index of graphene. Two single-layer graphene sheets are placed on the bottom waveguide with a 10 nm SiO_2_ gap. The electrical doping level (Fermi level, *E*_*F*_) of graphene is controlled by the tuning of the gate-voltage. So, if the doping level of the top graphene layer is adjusted to *E*_*F*_ = *E*_0_ eV, that of the bottom graphene layer is adjusted to *E*_*F*_ = −*E*_0_ eV. Therefore, since the two graphene layers act as active layers, the structure can enhance the effect of graphene. In the parallel-plate capacitor model, the required gate-voltage to adjust a certain *E*_*F*_ decreases as the distance between the graphene layers decreases.

Figure 1b illustrates a typical Λ configuration for the observation of EIT in an atomic structure. When the Λ system with a probe beam is illuminated with a strong control field, the population can go from |1⟩ to |2⟩ through two different paths: (A) |1⟩→|2⟩ (direct path) or (B) |1⟩→|2⟩→|3⟩→|2⟩ (circular path). EIT occurs because of the quantum destructive interference between the probability amplitudes for the two different paths (A) and (B), provided that the decay rate of |3⟩ is relatively smaller than that of |2⟩^47^. It is well known that the EIT-like transmission spectrum can be obtained by coupling high and low Q-factor resonant modes. The proposed photonic crystal structure (without graphene and SiO_2_ layers) excites high and low Q-factor modes simultaneously in the top and bottom waveguides, respectively. We note that the low- and high-Q modes in the proposed photonic structure correspond to the |2⟩ and |3⟩ states in the atomic structure, respectively. Our previous works reported the details in the phase matching condition to generate EIT-like transmission and related dispersion relation of the proposed structure^44,47^. By utilizing the high-Q modes that enable strong light-matter interactions, the modulation efficiency of the transmitted THz wave can be improved.

## Results and discussion

**Figure 2:**
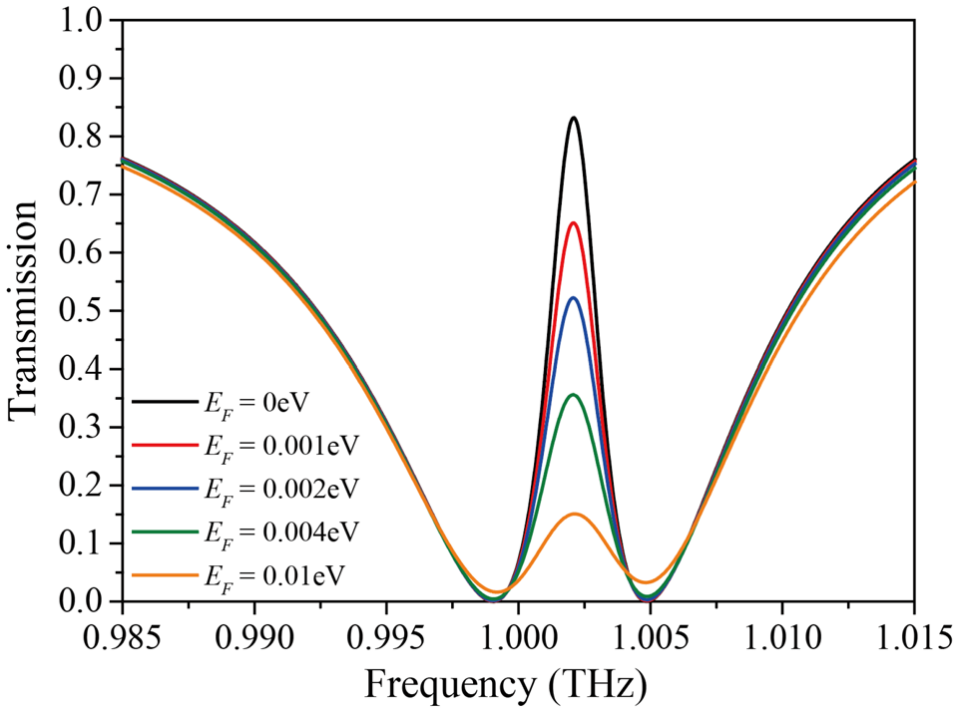
Transmission spectra for Fermi level variation from *E*_*F*_ = 0–0.01 eV. The period structure is *a* = 218 μm, and the width and height of the photonic crystal are *w*_pc_ = 0.7*a*, and *t*_pc_ = 0.2*a*, respectively. The thicknesses of the top and bottom waveguides are *t*_1_ = 0.25*a* and *t*_2_ = 0.361*a*, respectively, and the gap size is *g* = 0.8*a*.

Figure 2 presents the transmission spectra of the designed structure for the Fermi level variation from *E*_*F*_ = 0–0.01 eV when a THz wave is normally incident to the structure. The period, width, and thickness of the PC are assumed to be *a* = 218 μm, *w*_pc_ = 0.7*a*, and *t*_pc_ = 0.2*a*, respectively. The thicknesses of the top and bottom waveguides are *t*_1_ = 0.25*a* and *t*_2_ = 0.361*a*, respectively, and the gap size is *g* = 0.8*a*.

It is observed that the EIT-like transmission peak with a high Q-factor (~ 544) occurs at *f* = 1.002 THz, and the transmission peak decreases remarkably as the Fermi level increases at this frequency owing to the strong light-graphene interaction from the high Q-factor. In contrast, the transmission variation for the Fermi level variation is negligible at the frequency outside the transmission peak region owing to the weak light-graphene interaction.

**Figure 3:**
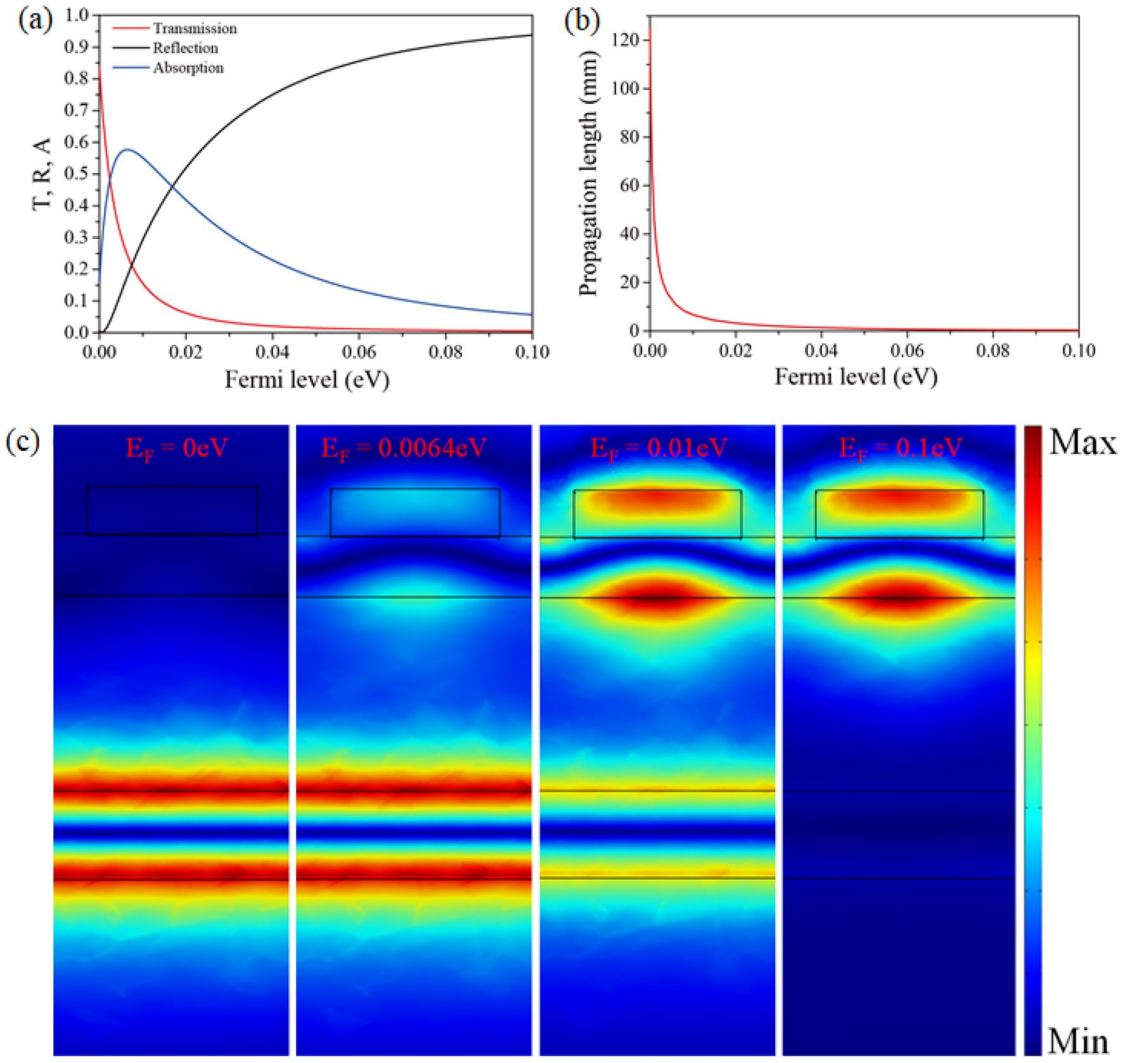
(**a**) Transmission, reflection, and absorption curves of the modulator at the EIT-like transmission frequency. (**b**) Propagation length of the bottom waveguide comprising a five-layer structure (air–SiO_2_ (*t*_2_)–graphene–SiO_2_ (10 m)–graphene–air). (**c**) Electric field distribution of the modulator for Fermi level variation from *E*_*F*_ = 0 eV, 0.0064 eV, 0.01 eV, and 0.1 eV, respectively.

The transmission, reflection, and absorption curves at the EIT frequency (*f* = 1.002 THz) are presented in Fig. 3a. At this frequency, the light propagating within the PC is strongly coupled to the bottom waveguide, and the coupled light propagates through the bottom waveguide for a long time, thereby resulting in highly enhanced light-graphene interaction. In other words, the EIT phenomenon significantly increases absorption using graphene. Here, the absorption is determined by the propagation loss of the bottom waveguide and coupling efficiency, which is the efficiency of power transfer from the top to bottom waveguides.

Figure 3b presents the propagation length (PL = 0.5/Im(*β*)) of the bottom waveguide for the Fermi level variation, which is calculated by the numerical analysis of the one-dimensional waveguide by solving Maxwell’s equations. *β* is the wave vector of the high-Q guided mode in the bottom waveguide comprising a five-layer structure (air–SiO_2_ (*t*_2_)–graphene–SiO_2_ (10 m)–graphene–air). As the Fermi level increases, the PL decreases because the imaginary part of graphene’s permittivity increases as the Fermi level increases. In addition, the PL decreases abruptly from *E*_*F*_ = 0–0.007 eV. This implies that the loss by graphene increases rapidly in this Fermi level range.

Furthermore, we illustrate the field distributions for the Fermi level variation in Fig. 3c. When the Fermi level is *E*_*F*_ = 0 eV, most of the light is coupled to the bottom waveguide, and the electric field is highly confined in the bottom waveguide. In contrast, as the Fermi level increases, less incident light is coupled to the bottom waveguide, and the electric field becomes confined within the top waveguide, which originates from the increase in the wave vector and mode mismatches. Hence the propagation loss decreases as the Fermi level increases. The absorption has a maximum value at *E*_*F*_ = 0.0064 eV. Below this Fermi level, the propagation loss is the dominant factor for absorption, while the coupling efficiency is the main determinant of absorption at higher Fermi levels.

**Figure 4:**
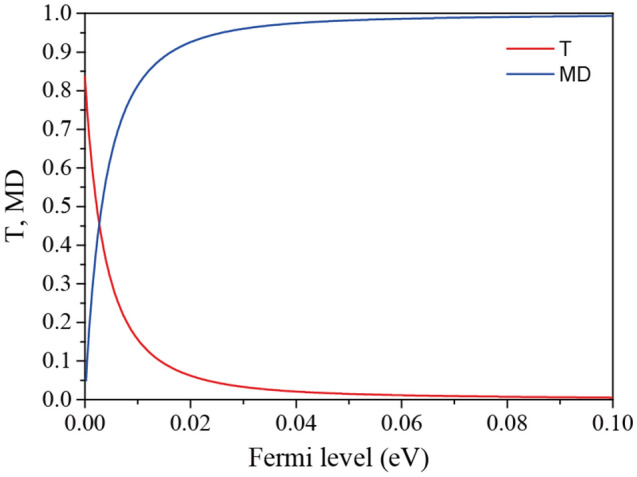
Transmission and modulation depth as a function of the Fermi level at the EIT-like transmission wavelength.

Figure 4 illustrates the transmission and modulation depth as a function of the Fermi level. The modulation depth is defined as *MD* = (*T*_*on*_–*T*_*off*_)/*T*_*on*_. If we assume the on- and off-states to be *E*_*F*_ = 0 and 0.1 eV, respectively, the proposed modulator achieves a high modulation depth of ~ 99.3% and an insertion loss of 16.5% with a small Fermi level shift of 0.1 eV. In addition, if we assume the off-state to be 0.05 eV, a modulation of ~ 98.2% can be achieved with a negligible Fermi level shift of 0.05 eV. Note that graphene-based modulators reported in Ref.^25–33^ require a minimum Fermi level shift of 0.2 eV. In addition, the maximum modulation depth among them is 87%. Compared to these modulators, the proposed modulator achieves significantly low energy consumption and high modulation depth simultaneously. In addition, the proposed modulator exhibits polarization-independent spectral response, which may be beneficial in practical applications.

It is important to discuss that fabrication process of the proposed structure. The 2D PC with a period of approximately 200 μm can be fabricated by conventional lithography techniques. Previous studies have reported that two graphene layers with a distance of a few nanometers can be realized on SiO_2_^48,49^. The proposed structure can be manufactured by a similar fabrication process. A graphene sheet on a copper film grown by CVD is transferred onto the SiO_2_ substrate using the standard wet-transfer technique^50^. Then, thermal evaporation is used to deposit metal for the electrode. Subsequently, a 10 nm SiO_2_ layer is deposited on the graphene layer using plasma-enhanced chemical vapor deposition (PECVD). The top graphene layer is transferred onto the SiO_2_ layer using the same process applied to the bottom graphene layer, followed by metal deposition for the electrode. Hence, the proposed highly efficient THz modulator structure can be realized by current fabrication technologies.

## Conclusion

We proposed a high-modulation depth, low-energy-consumption graphene-based modulator using the EIT-like transmission. The high Q-factor of the EIT transmission peak intensified the graphene absorption. The designed modulator achieved a significantly high modulation depth of 99.3% and a low insertion loss of 16.5% with a Fermi level shift of 0.1 eV. Furthermore, a modulation depth of 98.2% was achieved with a negligible Fermi level shift of 0.05 eV. In the proposed structure, the top waveguide with a 2D PC can be fabricated by conventional lithography techniques. In the bottom waveguide, the two-single layer graphene with a gap of 10 nm-SiO_2_ can be realized by current fabrication technologies. A graphene sheet on a copper film grown by CVD is transferred onto the SiO_2_ substrate using the standard wet-transfer technique. Thermal evaporation is used to deposit metal for the electrode. In subsequent, a 10 nm SiO_2_ layer is deposited on the graphene layer using PECVD. The top graphene layer and the electrode are realized by using the same process applied to the bottom graphene layer. A standard THz-time domain spectroscopy system can be employed in observing the tunable EIT-like transmission through the proposed structure.

## Methods

The conductivity of graphene was calculated using the Kubo formula expressed as^51–53^:

1 $$\begin{aligned} \sigma & = \frac{{2e^{2} }}{\pi \hbar }\frac{i}{\omega + i/\tau }\ln \left[ {2\cosh \left( {\frac{{E_{F} }}{{2k_{B} T}}} \right)} \right] + \frac{{e^{2} }}{4\hbar }\left[ {H\left( {\omega /2} \right) + \frac{4i\omega }{\pi }\int\limits_{0}^{\infty } {\frac{{H\left( x \right) - H\left( {\omega /2} \right)}}{{\omega^{2} - 4x^{2} }}dx} } \right] \\ H\left( x \right) & = \frac{{\sinh \left( {\hbar x/k_{B} T} \right)}}{{\cosh \left( {E_{F} /k_{B} T} \right) + \cosh \left( {\hbar x/k_{B} T} \right)}} \, \\ \end{aligned}$$

where *e*, *k*_B_, *T*, *ħ*, *ω*, and *E*_*F*_ denote the electron’s charge, Boltzmann’s constant, temperature, Planck’s constant, angular frequency, and Fermi level of graphene, respectively. The quantity of *τ* is the relaxation time defined as *τ* = *μE*_*F*_/*eν*_*F*_^2^, where *ν*_*F*_ represents the Fermi velocity (*ν*_*F*_ = 10^6^ m/s) and *μ* denotes the carrier mobility (*μ* = 10,000 cm^2^/V). From the conductivity of graphene, the permittivity of graphene can be obtained by

2 $$\varepsilon_{G} = 1 + \frac{i\sigma }{{\omega \varepsilon_{0} d_{G} }}$$

where *ε*_0_ and *d*_G_ represent the vacuum permittivity and thickness of graphene (*d*_G_ = 0.34 nm), respectively.

To calculate the transmission spectra, we adopted the periodic boundary condition for the *x*- and *y*-directions. In addition, we adopted a transition boundary condition (TBC) to model the graphene layer as a 2D plane in the geometrical domain. TBC represents a discontinuity in the tangential electric field due to the surface current density. Note that the identical transmission spectra can be obtained when the graphene layer is treated as the 2D sheet using the surface current density boundary condition. The 10 nm-SiO_2_ layer sandwiched with two graphene layers was divided by a 2.5 nm grid size in the calculations.

## Data Availability

The datasets used and/or analyzed during the current study available from the corresponding author on reasonable request.
